# 

**Published:** 1888-09

**Authors:** 


					﻿Volume LVII.I
SEPTEMBER, 1888.
I Number 3.

sM Bl fie*Al MK
|M miγn J hl B
a áKfil fli IIBI ^1N
EDITOR:
S. J. JONES, M.D., LL.D
Rand, McNally & Company, Printers.
1888.
Entered at the Post Office at Chicago, for transmission through the mails as second-class matter.
				

